# Real-time extended psychophysiological analysis of financial risk processing

**DOI:** 10.1371/journal.pone.0269752

**Published:** 2022-07-25

**Authors:** Manish Singh, Qingyang Xu, Sarah J. Wang, Tinah Hong, Mohammad M. Ghassemi, Andrew W. Lo

**Affiliations:** 1 MIT Laboratory for Financial Engineering, Cambridge, Massachusetts, United States of America; 2 Operations Research Center, Massachusetts Institute of Technology, Cambridge, Massachusetts, United States of America; 3 Department of Electrical Engineering and Computer Science, Massachusetts Institute of Technology, Cambridge, Massachusetts, United States of America; 4 MIT Sloan School of Management, Cambridge, Massachusetts, United States of America; 5 Computer Science and Artificial Intelligence Laboratory, Massachusetts Institute of Technology, Cambridge, Massachusetts, United States of America; 6 Department of Computer Science and Engineering, Michigan State University, East Lansing, MI, United States of America; 7 Ghamut Corporation, East Lansing, MI, United States of America; Vilnius University, LITHUANIA

## Abstract

We study the relationships between the real-time psychophysiological activity of professional traders, their financial transactions, and market fluctuations. We collected multiple physiological signals such as heart rate, blood volume pulse, and electrodermal activity of 55 traders at a leading global financial institution during their normal working hours over a five-day period. Using their physiological measurements, we implemented a novel metric of trader’s “psychophysiological activation” to capture affect such as excitement, stress and irritation. We find statistically significant relations between traders’ psychophysiological activation levels and such as their financial transactions, market fluctuations, the type of financial products they traded, and their trading experience. We conducted post-measurement interviews with traders who participated in this study to obtain additional insights in the key factors driving their psychophysiological activation during financial risk processing. Our work illustrates that psychophysiological activation plays a prominent role in financial risk processing for professional traders.

## Introduction

Several experimental studies in the field of behavioral economics have documented that emotional states (for example, fear, excitement, and stress) may influence the decision making of an agent [1–3]. In financial markets, sudden fluctuations in prices, changes in investor holdings, or other factors unknown to the agent may impact decisions. To better understand the biological and psychological mechanism of financial decision making under uncertainty, the emerging field of neurofinance has incorporated insights from psychology and neuroscience into theories of finance [4].

In previous studies, researchers characterized the psychophysiological (PP) activities of traders using functional magnetic resonance imaging (fMRI), hormonal level measurement, and physiological signals like heart rate, blood pressure, and skin temperature (reviewed in Section *Literature Review*). However, these techniques share the common limitation that, due to the equipment used to measure the physiological signals, the experiments are either conducted in laboratory settings rather than in the field, or the data collection procedure interferes with the day-to-day activities of traders. While controlled experimentation yields cleaner inferences, their findings lack the direct applicability to real financial settings which *in situ* experiments provide.

In this work, we conducted an *in situ* experiment and collected the physiological signals of 55 professional financial traders at a global financial institution during the entire trading day over a five-day period for each trader. Using an Empatica E4 wristband, we capture a variety of real-time physiological signals, such as heart rate, blood volume pulse, inter-beat interval, electrodermal activity, skin temperature, and three-dimensional accelerations of wrist motion, without interfering with the traders’ daily routines on their trading desks. This non-invasive experimental design offers the most realistic setting to date for analyzing the traders’ real-time PP activities during financial decision making.

The primary focus of our analysis was to measure the relation (if any) between a trader’s PP state and their financial decisions or market events, both contemporaneously and temporally. Since a trader’s PP state is not directly observed and must be inferred from the physiological signals, we measure each trader’s “psychophysiological (PP) activation”, defined as the collective deviation of physiological signals from their baseline values. Higher levels of PP activation indicates emotions such as excitement, stress, agitation, arousal, and irritation which elevate the physiological activities from the normal state. By analyzing the changes in the trader’s PP activation levels, we identify the circumstances in which such changes coincided with, or were caused by, financial transactions or market events. The key challenge was to accurately measure the levels of PP activation, and a major contribution of our study is the novel application of the Mahalanobis distance [5] on features extracted from physiological signals as a quantitative metric of PP activation.

There are two underlying assumptions of our analysis. First, financial decision making and risk processing under uncertainty trigger PP activation in professional traders. Second, the levels of the trader’s PP activation can be explained by multiple factors such as market fluctuations, the types of financial products traded, as well as the trading experience of each trader. We test four hypotheses related to these two assumptions:

- Traders monitor multiple market indices during working hours. Fluctuations in these market indices often represent trading signals to the traders and may affect the existing positions and real-time profit and loss (PnL) of the traders. We test the hypothesis that market fluctuations have causal influence on the PP activation of the traders.
- Traders who participated in our study have different lengths of trading experience ranging from 10 months to 25 years. As traders gain more trading experience, they may develop skills to manage their PP activation level when making risky financial decisions. Since we do not observe these skills, we hypothesize that the ability to manage one’s activation level is positively correlated with the length of one’s trading experience and test the statistical relationship between the trading experience and activation levels of the traders.
- Traders who participated in our study worked in different business divisions and traded different financial products. Since different financial products have different market volatility, dollar volume of transactions, and trading frequency, we expect the traders to have different patterns of PP activation depending on the type of their business division. We test the hypothesis that the traders’ PP activation is related to their business divisions or the financial products they trade.
- As traders engage in risky financial transactions, their PP activation levels may change due to the financial decision making process before, during, and after the transaction. The PP activation levels may also depend on other factors such as the number of transactions and the average dollar volume. We examine the change in traders’ PP activation before and after making a transaction and identify the period when the traders are the most highly activated. We test the hypothesis that the traders’ PP activation levels are related to their trading frequency and the average dollar volume of their transactions.

We found statistically significant relationships between the levels of PP activation and financial market movements, and that different traders monitored different market signals which, in turn, had causal influence on their PP activation levels. We found that traders with more trading experience had lower levels of activation. We also found a significant relationship between a trader’s activation level and the type of financial products being traded. For example, traders specializing in G10 rates, equities, commodity and foreign exchange had higher level of PP activation, while traders in securitized market generally had lower activation. Differences in the properties of the financial products lead to differences in traders’ decision making process, and hence to their PP activation levels. We also found that traders on average have the highest activation levels 15 to 25 minutes after making the transaction.

We conducted follow-up interviews with 14 of the 55 traders to review their individual results, and received useful feedback which helps us interpret their PP activation patterns in the context of their specific working environments. The interviews yielded additional factors which influence traders’ PP activation, such as the demand of the workday, social events, and their managerial responsibilities. We found that busier workdays and social events led to higher PP activation in some traders.

## Literature review

A major challenge in conducting neurofinance field studies is the faithful measurement of the trader’s real-time neural activities during live trading over extended periods of time [4, 6]. Most previous studies in the literature encountered an important tradeoff between measurement and environment, since measuring traders’ neural activities in real time required a controlled experimental setting. As a result, these studies typically used mock trading and simulated market events to replace the conditions of live trading. Many studies [7–10] used fMRI to continuously monitor participants’ neural activity during simulated trading exercises. Another study [11] designed simulated lottery games to study the effect of elevated cortisol levels on the degree of risk aversion of the participants.

While the controlled experimental setting provides a rigorous framework for data collection and analysis, several issues prevent one from applying the methodology and conclusions of these studies to professional traders engaging in real-world trading activities. First, it is difficult to perform neural activity measurements during live trading seamlessly without interfering in the normal work routine of the trader. The act of making the measurements may have a lasting impact on the participant’s mental state, which is difficult to calibrate and remove from the analysis. In addition, the neural activity profile of a participant (who may not be a professional trader) during mock trading [8, 11] is likely to be different from professional traders during live trading, since in the latter situation the traders incur real profit and loss (PnL) to their companies as a consequence of their decisions. Performing physiological measurements during live trading sessions often required non-disruptive measurement schemes at discrete points in time during or after trading hours rather than real-time monitoring. Lo *et al.* [12] used daily emotional-state surveys, and found that traders who react more intensely to their monetary gains and losses exhibited significantly worse PnL. Numerous studies [13–17] used saliva samples to measure traders’ intra-daily cortisol and testosterone levels and studied the relation between traders’ hormone levels and their trading performance measured by PnL. Although saliva sampling may faithfully reflect a trader’s PP state during live trading without causing much disruption to their normal work routine, it is difficult to perform such measurements continuously in time, and the studies cited above collected saliva samples twice or three times each day. These measurement schemes cannot effectively capture a trader’s real-time PP responses to transient market events and fluctuations in asset prices and market indices.

Lo and Repin [18] were the first to measure traders’ real-time physiology during live trading sessions. They also collected financial market data relevant to the traders’ financial decisions synchronously during the physiology measurements. The time series data allowed them to identify PP responses to transient market events and periods of high market volatility, though their study involved relatively few subjects (10 traders) and short periods of measurement (ranging from 49 to 83 minutes) when the physiological signals were collected from each trader.

In this study, we extended the line of research of [18] and collected physiological signals of 55 professional traders at a global financial institution. Data collection for each trader lasted five consecutive workdays over a week, and six to eight hours each day. The larger sample size and longer measurement periods allowed us to study the PP behavior of traders in a more unconstrained and natural setting. We used the Empatica E4 wristband to collect high-frequency physiology time series data without disrupting the traders’ normal work routine. We also collected relevant market data and financial transaction data of individual traders to analyze their PP responses to market fluctuations and financial transactions.

Another contribution of this study is our novel method for measuring the PP activation from physiological signals to capture psychological states such as excitement, stress and irritation. Higher activation is known to trigger psychological, behavioral, and physiological changes in the brain [19]. These changes influence different biological signals, such as electrocardiogram readings, blood volume pulse, electrodermal activity, heart rate variability, and skin temperature [20]. Since stress is an important cause of PP activation, the rich literature of stress detection from physiological signals may be applied to measure PP activation. Al-Shargie *et al.* [21] measured electroencephalography and functional near-infrared spectroscopy on the prefrontal cortex for stress assessment. Several studies [22–25] used features extracted from electrodermal activity to train stress detection machine learning models, while others [23, 26–28] analyzed heart rate variability for stress detection. In addition, photoplethysmography [29] and skin temperature [30] are also used for stress detection. Fernandez *et al.* [31] proposed a system which uses commercial devices to detect stress and alert in traders in real time, which is of direct interest to our purpose.

Previous experimental studies generally used artificial methods to elevate the activation levels in the subjects, and measured their biochemical markers or physiological responses synchronously [32]. This “ground truth” information of the activation patterns makes it possible to train machine learning models for PP activation analysis. However, such “ground truth” information is not available in our study since we are agnostic of the factors which elevate the traders’ activation. Instead, we propose a novel metric for PP activation using the Mahalanobis distance [5] of features extracted from physiological signals, which to the best of our knowledge has not been previously explored. This metric allows us to rigorously test the statistical relationship between PP activation and various aspects of financial decision making and risk processing, discussed in the next section.

## Materials and methods

To rigorously analyze how financial risk processing affects a trader’s PP state, we collected both financial transaction records and the real-time physiological signals (Table A in S1 Appendix) of 55 professional day traders at a global financial institution during their normal trading activities. Before data collection, we collected the demographic information of the traders such as gender, trading experience, and business division via surveys. To understand the impact of market fluctuations on the trader’s PP activation, we also acquired 10 intraday financial market time series (Table B in S1 Appendix) commonly monitored by the traders during the same period as we measured their physiology. The details of data collection and preprocessing procedures are described in Sections A to F in S1 Appendix.

### Measuring PP activation

From the physiological signals of the traders, we constructed a synthetic metric to characterize each trader’s PP activation, defined as the physiological response to emotions like excitement, stress, and irritation. We chose to measure PP activation since it is ubiquitous in the context of financial risk processing. Events such as high market volatility, sudden gain or loss in the trader’s portfolio, or receiving instructions from clients to place transactions may induce elevated activation in traders. Previous studies showed that a high level of activation is associated with reduced performance [33, 34]. In addition, prolonged periods of high activation have an adverse impact on health [35, 36]. We extract PP activation from the physiological features in the following way:

- Heart Rate Variability (HRV): HRV measures the variations in the beat-to-beat intervals. Inspired by [28], we calculated four features from the raw HRV signal to measure activation: mHR, the average number of beats per minute; mRRi, the average value of the interbeat (RR) interval; SDNN, the standard deviation of the RR interval; and RMSSD, the root mean square sum of the successive interbeat interval difference. All features are calculated as time series using a 5-minute trailing window, similar to [28].
- Electrodermal Activity (EDA): EDA measures the electrical conductivity of the skin. Elevated activation increases the skin temperature and sweating, which in turn changes the EDA. We calculated three features from the raw EDA signal to measure activation: mAmp, the average of the raw skin conductance signal; Slope, the average of the absolute first difference of the skin conductance signal; and Events, the number of times the EDA signal increases more than 0.05*μS* in less than 5 seconds. These features are inspired by [24].
- Blood Volume Pulse (BVP): BVP is measured by a photoplethysmogram (PPG) sensor, which uses optical technique to measure the blood flow rate and blood volume. Blood pressure is then estimated using BVP readings [37]. Elevated cardiovascular activity due to higher activation changes the BVP. We calculated three features from the raw BVP signal to measure activation: the average of the absolute value of the BVP; the minimum value of BVP, and the maximum value of BVP. These features are inspired by [24].
- Skin Temperature (TEMP): TEMP generally increases during periods of high activation. The changes in skin temperature depend on the specific body region being measured [30] and changes in room temperature. We assume that changes in room temperature occur much more slowly than the sudden onset of events that lead to high activation. We compute the instantaneous rate of change in TEMP to capture the change in levels of PP activation.

Once we extracted these features as a column vector $y_{t}\in R^{d}$ at each time *t* for a trader, we computed the aggregate measure of PP activation using the Mahalanobis distance *d*_*M*_ [5],

$$\begin{array}{c} d_{M}\left(y_{t}\right)=\sqrt{{\left(y_{t}-\mu_{t}\right)}^{T}\Sigma_{t}^{-1}\left(y_{t}-\mu_{t}\right)} \end{array}$$ (1)

where the column vector $\mu_{t}\in R^{d}$ and matrix $\Sigma_{t}\in R^{d\times d}$ are the rolling sample mean and covariance matrix computed using *y*_1_, …, *y_t_*. The superscript *T* denotes vector transposition. We use the rolling mean and covariance to prevent the look-ahead bias of computing PP activation at time *t* using signals measured in future time *t*′ > *t*. The Mahalanobis distance captures the trader’s PP activation as the collective deviation of physiological activities *y*_*t*_ from their baseline state *μ*_*t*_ and adjusts for the covariance between the components of *y*_*t*_, which tend to be highly correlated if they are extracted from the same underlying physiological signal measured by the wearable device (shown in Table G of S1 Appendix).

Using the Mahalanobis distance, we computed five different measures of PP activation: the overall PP activation (using features extracted from all physiological signals), and the individual HRV, EDA, BVP, and TEMP activation (using features extracted from one physiological signal) for every trader. In the subsequent analysis, we focus on the overall PP activation since it captures the collective excitation of all physiological activities simultaneously.

Next, we identify the time periods when each trader is under mild and extreme levels of PP activation by measuring their deviations from mean. We label the trader as “mildly activated” at time *t* if her PP activation *d*_*M*_(*y*_*t*_) satisfies *d*_*M*_(*y*_*t*_) > *μ* + 1.5*σ* where *μ* and *σ* denote the mean and standard deviation of her PP activation during the trading day. Details of computing *μ* and *σ* are provided in Section F in S1 Appendix. Similarly, we label the trader as “extremely activated” at time *t* if *d*_*M*_(*y*_*t*_)>*μ* + 3*σ*. We choose thresholds 1.5 and 3 based on qualitative assessment of the magnitude of mild and extreme activation. Future extensions of our work may use data-driven methods to identify periods of mild and extreme activation for each trader.

Finally, we use three aggregate metrics to characterize the PP activation patterns of each trader: activation proportion, activation length, and average activation. Activation proportion measures the percentage of time when the trader was at a mild or extreme level of activation during a trading day. Traders with higher activation proportion tended to be more susceptible to elevated activation. Activation length measures the average duration of the mild or extreme activation periods of a trader. Traders with longer activation length tended to remain activated for longer periods of time after the sudden onset of events which triggered activation. Average activation measures the average value of PP activation over the trading day and reflects both activation proportion and length. Traders with higher activation proportion or length also have higher average activation. These aggregate measures allowed us to directly compare the PP activation patterns across different traders and trading days. The definition and computation of activation proportion and activation length are described in Section F in S1 Appendix.

### Activation level attribution regression

To identify the relationship between individual factors and the PP activation of the traders, we perform the following regression:

$$\begin{array}{c} y_{i,t}=\alpha +\beta *bis_{i}+\gamma *gen_{i}+\delta *amt_{i,t}+\theta *exp_{i}+\zeta *numT_{i,t}+\eta *vol_{i,t} \end{array}$$ (2)

where *y*_*i*,*t*_ denotes the activation metric of the trader *i* on day *t*, *bis*_*i*_ the business division (one-hot encoded), *gen*_*i*_ the gender (male/female), *amt*_*i*,*t*_ the average dollar value of the transactions by trader *i* on day *t*, and *numT*_*i*,*t*_ the number of transactions by the trader on day *t*. Since traders’ activation may also be correlated with market volatility on different days, we also include *vol*_*i*,*t*_, the vector of volatility of different market indices on day *t*. The right-hand-side explanatory variables of the regression were extracted from trader survey, financial market indices, and traders’ transaction data. We use the average activation as the left-hand-side dependent variable *y*_*i*,*t*_ since it reflects the other activation metrics (proportion and length).

### Granger causality test

We test whether there is Granger causality relation between fluctuations of market indices and trader’s PP activation. Specifically, let *y*_*t*_ and *m*_*t*_ denote the minute-level time series of a trader’s PP activation and a market index on a trading day. Since the activation time series is measured with 1Hz (higher frequency than market indices), we perform frequency averaging on *y*_*t*_ to get the minute-level activation time series ${\bar{y}}_{t}$. To account for non-stationarity in the time series, we perform the Granger causality test on the first difference $\Delta {\bar{y}}_{t}={\bar{y}}_{t}-{\bar{y}}_{t-1}$ and *Δm*_*t*_ = *m*_*t*_ − *m*_*t*−1_. For each trader and each market index on a given trading day, we test the null hypothesis that Δ*m*_*t*_ does not Granger-cause $\Delta {\bar{y}}_{t}$. The Granger causality test is performed with the sum of squared residual (SSR) test with chi-squared distribution using the **statsmodels** package in the SciPy library and a lag of 10 minutes. To correct for multiple hypothesis testing across all traders, we perform the Holm-Bonferroni correction for each market index with significance level 0.05.

### Activation around financial transactions

We analyzed the pattern of trader’s PP activation around financial transactions. Specifically, we computed the evolution of PP activation using a rolling window before and after placing a trade in the following way:

- We first calculated the z-scores of PP activation *z*_*t*_ so that the activation levels may be consistently compared across different time periods and traders.
- Next, for a transaction at time *l* placed by the trader, we calculated the mean values of *z*_*t*_ in the windows [*z*_*l*−30*min*_, *z*_*l* − 25*min*_], [*z*_*l* − 25*min*_, *z*_*l* − 20*min*_], …, [*z*_*l* + 20*min*_, *z*_*l* + 25*min*_], [*z*_*l* + 25*min*_, *z*_*l* + 30*min*_]. This computed the time series of activation averaged over 5 minute windows in 30 minutes timeframe before and after performing the transaction.
- Finally, for each trader in a trading day, we averaged the rolling window calculation results across all transactions made by this trader on this day. We computed the evolution of PP activation around a transaction by averaging the results across all traders and trading days.

### Individual trader meetings

The 55 traders who participated in our study had diverse levels of trading experience and traded a variety of financial products. Since the physiological signals were recorded during their normal working hours, their PP activation levels may have been affected by idiosyncratic sources that were exogenous to their trading activities and unknown to us. To investigate these idiosyncratic sources of PP activation and relate them to our findings, we invited all participating traders to meet individually with us after we completed the PP activation analysis. Due to traders’ availability, 14 of 55 traders participated in the individual meetings. During each meeting, we presented the individual findings to the trader and received the feedback from the trader. We applied the insights from these trader meetings to identify additional sources of PP activation.

### Ethics statement

The study was approved by the following IRB/ethics committee: MIT Committee on the Use of Humans as Experimental Subjects (COUHES). The study approval number is 0403000144 and the written consent was obtained prior to the study.

## Results

We present the analysis results of traders’ PP activation and investigate the different sources that may cause elevations in PP activation levels. We discuss both general patterns of trader’s PP activation observed across all traders and idiosyncratic characteristics specific to individual traders.

### Variation in trader’s activation levels

**Fig 1:**
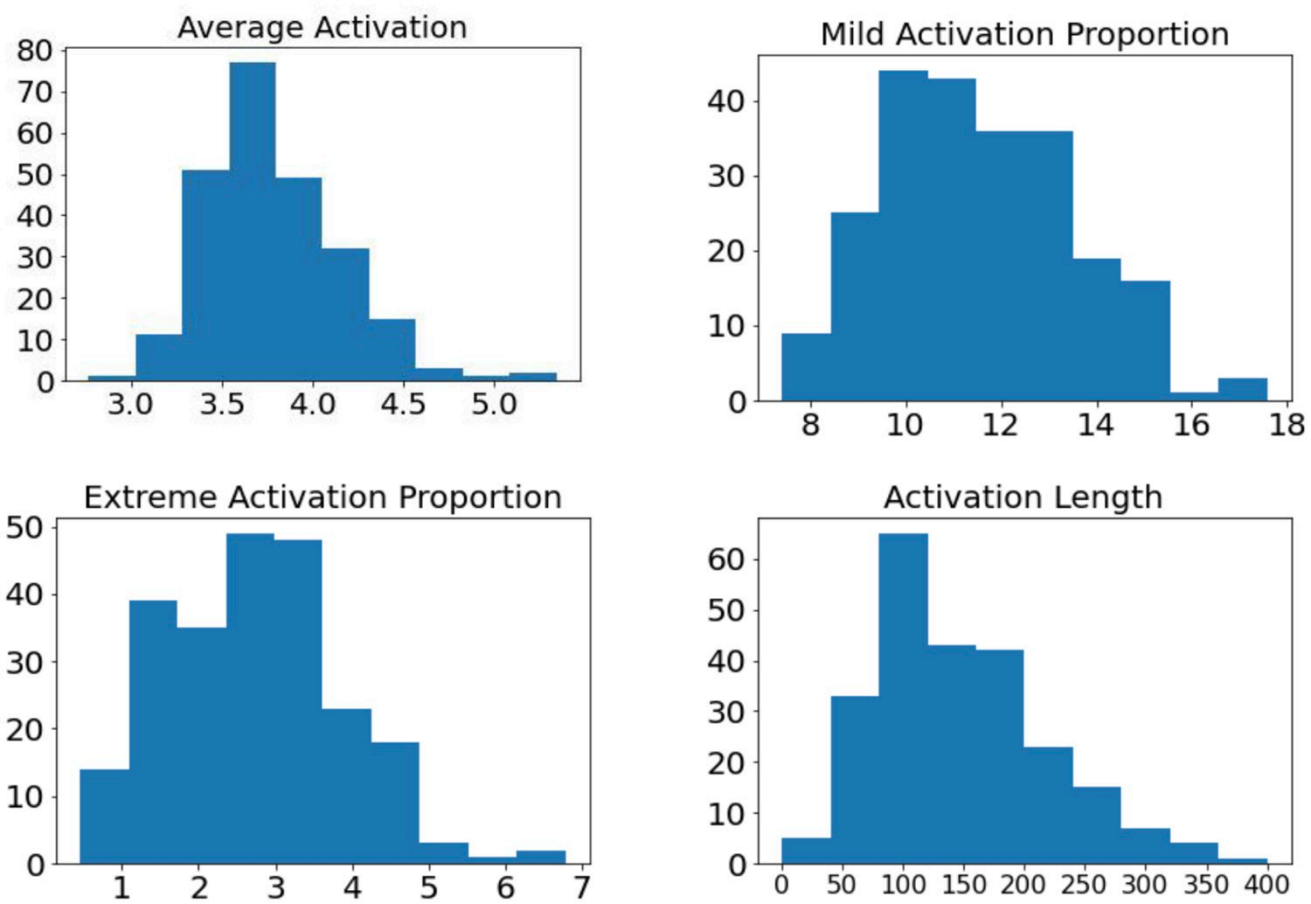
Histograms of four PP activation metrics. The y-axis represents the number of trader-days and the x-axis represents the activation metric values. We observe considerable variations in PP activation levels across traders. Average activation is a dimensionless quantity. Mild and extreme activation proportions are measured in percentages (%). Activation length is measured in seconds (s).

We analyzed the variation in PP activation patterns across different traders as reflected by four activation metrics: average activation, mild activation proportion, extreme activation proportion, and activation length. Fig 1 shows the histograms of these metrics for all traders. A trader’s PP activation metric on each trading day is counted separately in the histogram to account for inter-day variation and there are a total of 242 trader-day pairs in each histogram. The summary statistics of each histogram is shown in Table 1.

**Table 1:** Summary statistics of four PP activation metrics. Average activation is a dimensionless quantity. Mild and extreme activation proportions are measured in percentages (%). Activation length is measured in seconds (s).

Metric	Min.	Max.	Mean	Std. Dev.
Average Activation	2.76	5.34	3.79	0.37
Mild Activation Proportion (%)	7.42	17.57	11.54	1.99
Extreme Activation Proportion (%)	0.45	6.78	2.71	1.12
Activation Length (s)	26	1031	152	94

We observed significant variation in all PP activation metrics across the traders. Several traders were at mild activation for 17% of working hours on certain days, while others were at mild activation for less than 8% of the time. Similarly, some traders returned to their normal activation levels in less than 30 seconds from the onset of elevated activation, while others took as long as 17 minutes. The detailed statistics of PP activation metrics for each trader is summarized in Table E in S1 Appendix. The significant variation in PP activation metrics can partly be ascribed to the diverse characteristics of the traders in various aspects, which motivates us to investigate the systematic and idiosyncratic factors which lead to the variation in PP activation levels.

### Factors influencing PP activation

We performed the regression (Eq 2) to identify the relationship between different factors and the PP activation of traders. The dependent variable of the regression *y*_*i*,*t*_ is the average PP activation of trader *i* on day *t*. The correlations between the covariates of the regression are summarized in Table F in S1 Appendix.

#### Gender

We do not find statistically significant relationship between the average PP activation and the gender of a trader (difference between the regression coefficients of male vs. female: −0.02, p-value: 0.83).

#### Trading experience

We find that traders with more trading experience have lower average PP activation (coefficient: −0.02, p-value: 0.01). The possible explanation is two-fold: traders may become more adept at making risky financial decisions under uncertainty with little emotional fluctuations or may acquire certain skills over time to actively manage their PP activation levels during financial risk processing.

#### Dollar volume, number of transactions, and market volatility

The average dollar volume and number of transactions have positive regression coefficients 0.02 (p-value: 0.26) and 0.4*e* − 4 (p-value: 0.47), respectively. While we observed an overall positive correlation between the dollar volume, number of transactions and the average PP activation of the traders, additional factors of their trading activities might further influence whether the transaction causes high or low PP activation and lead to the large p-values. During individual trader interviews, we found that traders’ PP activation highly depends on type of transaction (via a client’s order or the trader’s own decision) and the changes in risk exposure as a result of the transaction. Similarly, the regression coefficients of volatility of different market indices are not statistically significant. This motivates us to analyze the time series characteristics of market fluctuations and traders’ PP activation via the Granger causality test discussed later.

#### Other factors

We also found statistically significant relations between the trader’s average PP activation and the type of financial products traded. A detailed discussion of these results is provided in Section H in S1 Appendix. During individual trader meetings, traders reported additional idiosyncratic factors that influenced their PP activation, including whether they had a busy schedule, their managerial responsibilities, and even the anticipation of the social events after work. These idiosyncratic factors were not measured in our experiment and may contribute to the variation in traders’ PP activation unexplained by the regression.

### Market fluctuations have causal impacts on traders’ PP activation

Typically, each trader manages a portfolio of many active positions and monitors a variety of market indices and events during the trading hours. It is reasonable to expect that increased volatility in market indices relevant to their portfolio values may cause the traders’ PP activation levels to become elevated. We tested the null hypothesis that market fluctuations do not have causal influence on traders’ PP activation via the Granger causality test, with Holm-Bonferroni correction at 5% significance level.

To test whether the observed Granger causality relations between market index and PP activation are statistically significant, we plot the distribution of p-values for each pair of PP activation and market index in Figure D in S1 Appendix. Under the null hypothesis *H*_0_ that the market index does not Granger-cause traders’ PP activation, the distribution of p-values of Granger tests should follow a uniform distribution on [0, 1]. We perform a one-sample Kolmogorov–Smirnov test and reject the null hypothesis *H*_0_ for six of the eight market indices (Credit Default Swap Index Investment Grade (IG), Credit Default Swap Index High Yield (HY), S&P 500 E-mini Futures Price, 10Y US Treasury Futures Price, 5Y US Treasury Futures Price and Crude Oil Futures Price) at significance level 0.05.

The number of statistically significant Granger causality relations between market fluctuations and traders’ PP activation is shown in Table 2. We observe that the two credit default swap (CDS) indices were the most common sources of elevated PP activation for the traders. Since CDS contracts act as insurance against credit defaults, it is reasonable that fluctuations in CDS indices have systematic impact on multiple financial products and elevate the PP activation levels among the largest number of traders.

**Table 2:** The number of statistically significant Granger causality relations between each market index and traders’ PP activation, with Holm-Bonferroni correction at 5% significance level.

Market Index	Number of significant GC relations
Credit-Default Swap Index IG	22
Credit-Default Swap Index HY	19
USD Index	3
10Y US Treasury Future Price	3
S&P 500 E-mini Future Price	4
5Y US Treasury Future Price	6
Crude Oil Futures Price	4
VIX Futures Price	7

### Financial transactions elevate traders’ PP activation

Traders regularly engage in financial transactions which induce considerable risks to their PnL performance. Real-time financial risk processing during a transaction can be a major source of high PP activation for the traders. Since transactions are placed at irregular points in time, we performed an event study to investigate the changes in trader’s PP activation levels before and after a transaction, as described in *Methods and Materials* Section.

**Fig 2:**
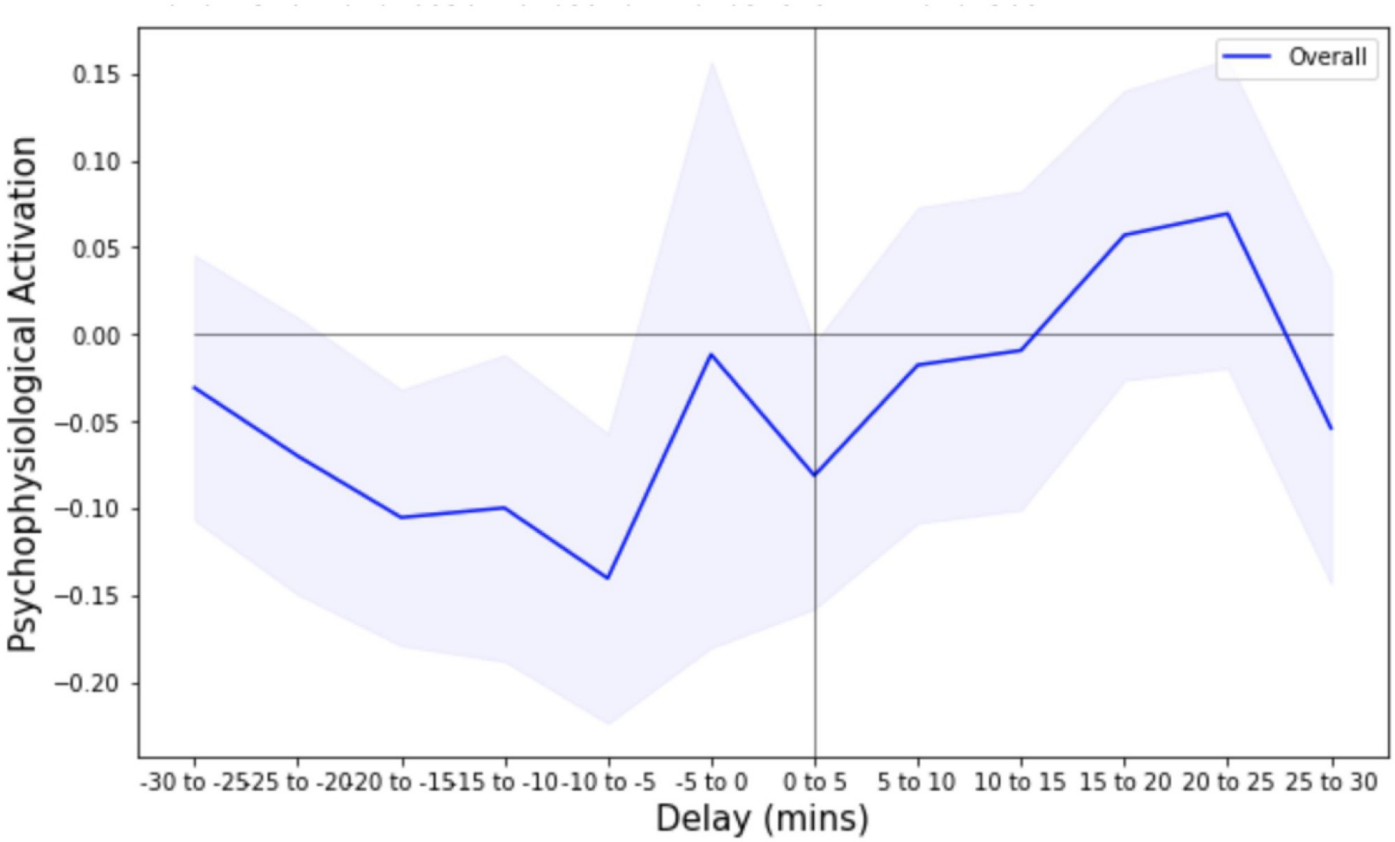
Evolution of trader’s PP activation around a transaction. The shaded region corresponds to the 95% confidence band. The x-axis is the time difference (in minutes) between the transaction time, defined as time 0, and measurement time. The y-axis is the average PP activation value. We observe that traders have the highest PP activation during 15 to 25 minutes after the transaction, as well as a local peak 5 minutes before the transaction.

Fig 2 shows the evolution of the average PP activation levels around a transaction, where the results are averaged across all traders. The vertical axis represents the average PP activation and horizontal axis indicates five-minute intervals around the transaction, where the transaction time is defined at *t* = 0. The left half of the figure corresponds to PP activation levels before the transaction, and the right half to those after. To account for the variation across traders, we also plot the 95% confidence band of PP activation.

We observe that traders tended to have the highest PP activation in the time window of 15 to 25 minutes after the transaction. We also observe a local peak of high PP activation 5 minutes prior to the transaction. During individual trader interviews, traders pointed out that their PP activation levels depended on the risk exposure of the transaction, as well as whether the transaction was placed via a client’s order or per the trader’s own discretion. In addition, traders mentioned that it usually takes 15 to 30 minutes after a transaction to confirm whether the transaction is executed as intended and to observe its effect on the trader’s PnL. This anticipation effect partly explains the elevated PP activation levels 15 to 25 minutes after transaction, shown in Fig 2.

## Discussion

Our analysis confirms that affect, as evidenced by the fluctuations of PP activation due to market fluctuations or financial transactions, plays a prominent role in the financial risk processing and decision making process for professional traders in their daily trading activities [18]. Our results provide contextual support for the growing literature of affective science investigating the relations between emotions and decision making [38–40] in the case of professional traders, whose profession requires them to make rational decisions under large uncertainties and maximize their payoffs.

Our study also illustrates the feasibility of conducting large field experiments in affective sciences with non-disruptive physiological measurement and the rich insights one can extract from this data using appropriate statistical techniques. For example, by utilizing the time series properties of PP activation, we overcome the challenge of performing causal inferences with observational data and demonstrate the causal relationship between certain market indices and traders’ PP activation. The novel use of Mahalanobis distance to capture the aggregate PP activation from individual physiological signals may be widely applied in many other contexts in this field.

Our analysis has several limitations to be addressed in future works. First, while we observed the presence of affect in financial risk processing, our analysis does not distinguish between positive and negative affects or investigate whether affect constitutes a beneficial or adverse driver of the traders’ financial performance. Previous studies revealed that positive affect leads to improved decision making [41] while negative affect such as fear and anger distorts the perception of risk [42] and causes myopic decisions [43]. During individual trader meetings, 12 of the 14 traders pointed out that they typically experienced high levels of activation when their real-time trading performance (measured by PnL) exhibited losses or fluctuations. Future study may analyze the relation between traders’ PnL performance and their PP activation under various market conditions using appropriate statistical or machine learning techniques [44]. Using such findings, one may even quantify the impact of affect on the trader’s PnL and prescribe strategies for traders to actively manage their affect and improve their trading performance.

In addition, while the field experiment design allows us to faithfully measure the physiological and affective state of professional traders in their natural working environment, it also prevents us from observing and controlling all the factors which influence the trader’s affect. As a result, our analysis does not make the important distinction between integral emotion (the emotion induced by financial risk processing which is our main interest) and incidental emotion (the emotion carried over from non-trading situations such as performing managerial tasks or participating in work meetings) [39]. This limits the interpretation of our findings since previous studies showed that incidental emotions affect risk perception [45] and induce bias on the financial decisions [46]. Future studies may improve the experimental design by asking participants to wear the measurement device only during trading activities or report the time periods when they are mainly occupied with non-trading activities. While this improves the statistical inference, researchers must also ensure that participants adhere to the experiment protocols.

Finally, from an evolutionary biology perspective, professional traders must adapt to their highly competitive, dynamic and uncertain working environment where significant portions of their compensation depend on their PnL. It is natural to hypothesize that the biological and psychological mechanisms of financial risk processing are markedly different between professional traders and the rest of the population [47]. Another interesting extension of our work is to conduct a similar experiment with a control group of subjects with little or no trading experience. In this way, one can determine the unique characteristics of professional traders’ cognitive and emotional faculties which enable them to thrive in the highly dynamic and uncertain working environment.

## Conclusion

We conducted a large field experiment and measured the real-time physiological signals of 55 professional traders during their normal trading activities over a five-day period. Using a novel metric of PP activation based on the Mahalanobis distance, we found large variations in PP activation across traders. We showed that the PP activation is correlated with and influenced by multiple factors, such as market fluctuations, financial transactions, as well as trader’s experience and types of financial products traded. Our analysis confirms the prominent role of affect in financial risk processing for professional traders. Future studies may analyze the impact of traders’ affect on their trading performance and the difference in affect between professional traders and subjects with no trading experience when making risky decisions under uncertainty.

## Supporting information

S1 Appendix (ZIP)
