# Supplementary material for: Real-time extended psychophysiological analysis of financial risk processing
Source: PLoS One. 2022 Jul 25;17(7):e0269752. doi: 10.1371/journal.pone.0269752 (PMC9312384; doi:10.1371/journal.pone.0269752)
Supplement: S1 Appendix — (ZIP) [file pone.0269752.s001.zip › S1.pdf]

Real-time extended psychophysiological analysis of financial risk processing

Manish Singh[1,3,5], Qingyang Xu[1,2], Sarah J. Wang[1], Tinah Hong[1], Mohammad M. Ghassemi[6,7], Andrew W. Lo[1,2,3,4,5*]

1 MIT Laboratory for Financial Engineering, Cambridge, Massachusetts, United States of America
2 Operations Research Center, Massachusetts Institute of Technology, Cambridge, Massachusetts, United States of America
3 Department of Electrical Engineering and Computer Science, Massachusetts Institute of Technology, Cambridge, Massachusetts, United States of America
4 MIT Sloan School of Management, Cambridge, Massachusetts, United States of America
5 Computer Science and Artificial Intelligence Laboratory, Massachusetts Institute of Technology, Cambridge, Massachusetts, United States of America
6 Department of Computer Science and Engineering, Michigan State University, East Lansing, MI, USA
7 Ghamut Corporation, East Lansing, MI, USA

* Corresponding Author: alo-admin@mit.edu

Supporting information

A. Market Efficiency

Efficient Market Hypothesis It states that security prices reflect all the available information. It is based on the standard economic paradigm of rational expectations postulates that all financial decision makers are rational agents who maximize their expected utility functions under all market circumstances [1]. However, this paradigm does not consider the role of psychophysiological state in financial risk processing of traders. In recent decades, the rational expectations paradigm has been challenged by empirical observations in the behavioral finance literature.

Adaptive Market Hypothesis It combines the principles of the efficient market hypothesis with behavioral finance to explain the deviations from the market efficiency [2]. It points out that investors are not always rational and security prices do not always trade at the fair value. It incorporates the psychological and behavioral characteristics of traders to explain deviations from rationality.

B.Physiological Data Collection

Subjects: Fifty-five professional traders working at a global financial institution participated in this study. The traders were divided into seven groups according to their trading desks. Six groups were based in the institution's headquarters, and one in a regional office. The traders were recruited by the financial institution and varied in age, gender, trading experience, and types of financial product they traded. Each group of traders typically belonged to the same trading desk and specialized in a particular type of financial products, such as municipal bonds, mortgage-backed securities, equities, and power and natural gas.

Physiology Data Collection Procedure The physiology data collection required seven weeks, and took place between March 25, 2019, and June 21, 2019. Each group of traders recorded their physiological waveforms via the Empatica E4 wristbands over five consecutive workdays (with a few exceptions). On the Friday before each week of data collection, the study team met with the traders who would participate in the following week to explain the data collection procedure and obtain their informed consent. Each trader received written instructions to wear the Empatica E4 wristband (as shown in Figure A) on the wrist of their non-dominant side from the time they arrived at the office until the end of the workday, Monday to Friday of their week. The collected physiological waveforms were downloaded to a secure device by the study team at the end of Monday, Wednesday, and Friday during each week. To ensure the anonymity of the traders, each trader was assigned a participant ID in the format of "[wristband serial #]_[week #]".

C. Market and Financial Data Collection

Market Time Series Data: To understand how real-time fluctuations of different market sectors affect trader's psychophysiological activation, we acquired data for 10 commonly used market indices and futures prices from three sources, as shown in Table B. The market time series data was recorded during the same period as the physiology data collection (between March 25, 2019, and June 21, 2019). We defined the average of bid and ask prices as the futures prices.

Financial Trading Data: To understand how trading affects the levels of psychophysiological activation of each trader, we acquired the financial trading records of all traders who participated in our study from the global financial institution. The features of the financial data we used included trade price, quantity, notional amount, execution timestamp, and the lifecycle state of the trade, as well as the market sector and type of financial product being traded.

The number of transactions made by traders in each week is shown in Table C. We observe significant variation in the number of transactions across different weeks. In particular, traders in week 5 made many more transactions than the others. In our feedback session, we learned that most of the traders in week 5 used trading algorithms to place these transactions automatically.

D. Trader Survey We asked traders to fill a survey during the trading days that included multiple questions. Some of them are years of trading experience, highest degree or level of school completed, gender, average number of transactions placed, business division they were part of.

E. Data Preprocessing

Preprocessing Physiological Waveforms: The physiological waveforms collected by the Empatica wristband (Table A) are measured in different frequencies. To reduce the measurement noise and align different physiologies in a multivariate time series with the same frequency, we first downsampled each raw physiological waveform to 1Hz by averaging the measurement values within each second. In our subsequent analysis, we removed the wrist accelerations (ACC) due to large irregularities which remained even after smoothing to 1Hz.

The inter-beat interval (IBI) measures the length of the time interval between two successive heartbeats, and does not have a regular frequency. Due to a measurement limitation of the Empatica E4 wristband, the IBI measurements for most traders were missing. To address this issue, we computed an average IBI time series using the instantaneous heart rate, HR_t: $IBI_t = 60/HR_t$.

Average IBI: Empatica E4 wristband needs 10 seconds of data for the heart rate calculation. Hence, IBI values obtained using instantanous heart rate is IBI averaged over 10 seconds. Hence, IBI_t values used in this analysis are average IBI values. When average IBI values are compared to the instantaneous IBI (when instantaneous IBI is available for longer durations), the mean squared error is 0.011 seconds and the error is considerably small as compared to the IBI values (0.6-1 second). In our analysis average IBI is one of the signal used for heart rate variability feature extraction that in turn was used for activation levels computation. We observed statistically significant relationships between activation values computed using average IBI and market, trading signals.

Remove Inactive Physiological Waveforms: We performed a visual inspection of each trader's physiological waveforms. We observed that 13 out of 55 traders had inactive periods in their physiology measurements when their BVP and EDA waveforms remained identically zero, as shown in Figure B (A). Since 8 out of these 13 traders showed inactive periods after 5PM ET, we hypothesize that the inactive periods were due to traders not turning off their wristbands as they left work.

Since no meaningful physiology data was collected during these inactive periods, it was necessary to identify and remove them from our analysis. To achieve this, we inspected each trader's physiological waveforms, manually labeled the start and end time points of each inactive period, and removed these inactive periods from the subsequent analysis.

Aggregate Block transactions In the financial trading data, we observed many instances where multiple transactions, each recorded with a unique trade ID, occurred within one second. Since it is highly unlikely that these transactions were placed independently by the trader, we consulted the global financial institution and learned that these transactions in fact belonged to one multi-leg "block trade" placed by the trader, but were recorded as individual transactions in the trading data. However, no feature in the trading data indicated whether two individual transactions belonged to the same block trade.

To aggregate the individual transactions back to the original block transactions, we adopted a simple heuristic: If the trader placed two consecutive transactions within one second, we assumed that they belonged to the same block trade. The number of aggregated block transactions is illustrated in Table D for three representative traders. We also validated the results with the number of transactions self-reported by the traders in their survey responses (column 5 in Table D).

In most cases (as shown in Table D), the number of aggregated block transactions agrees well with the value self-reported by the traders in their survey responses. However, we should caution that in a few cases, the number of block transactions is significantly above the self-reported values (which may not be accurate). Overall, this heuristic produced a reasonable approximation to the original multi-leg block transactions.

Align Market Time Series The time series of the futures prices (Table A) were recorded at the timestamps when the transactions were executed in the market. As a result, these timestamps are irregular and do not form a time series with a uniform frequency. To perform a statistical analysis requiring an evenly spaced time series (such as a Granger causality test), we aligned the market time series, m_t, to minute-level m_t^{align}. We used minute-level data since the USD index was recorded at the beginning of each minute. We defined the aligned time series value m_t^{align} at minute t as the time series value observed closest to and no later than time t, i.e. $m_t^{align} := m_\tau$ where $\tau := max_s\{s : s \leq t$ and m_s was observed$\}$. For notational simplicity, we use m_t to denote the market time series aligned to minute level in the subsequent analysis.

F. Activation Feature Extraction

Activation Proportion Once we obtained a time series of activation for each trader, we were interested in finding the regions of high activation. These regions of high activation are characterized by large deviations from the mean of the distribution. To find these regions, we used heuristics that are generally used for measuring deviations from mean, specifically choosing those that are dynamic and with a smaller number of tunable parameters.

- $arousal_t > mean(arousal)_{day} + \alpha * std(arousal)_{day}$: If the value of activation deviates from the mean value of arousal over the day, then it is considered an outlier. The value of α was set to 1.5 for periods of mild activation, and 3.0 for extreme activation.
- $arousal_t > mean(arousal)_{t:t-60min} + \alpha * std(arousal)_{t:t-60min}$: If the value of arousal deviates from the mean value of arousal calculated over a rolling window of 60 minutes, then it is considered an outlier. The value of α was set to 1.5 for periods of mild activation, and 3.0 for extreme activation.

The second heuristic is adaptive over time. Due to its adaptive nature, however, it may miss small outliers next to extreme outliers that may be captured by the first method. Hence, a period is considered high activation when it is flagged by either of the above methods. Finally, we computed the activation proportion for each trader, defined as the percentage of active trading time when the trader is at high activation. We calculated two variants of this proportion:

- Mild activation proportion: this is defined as the percentage of active trading time when the trader is under mild to high psychophysiological activation. For its calculation, the value of α was set to 1.5 in the outlier detection method. This metric captured periods of mild to high activation in traders.
- Extreme activation proportion: this is defined as the percentage of active trading time when the trader is under extremely high activation. For its calculation, the

value of α was set to 3.0 in the outlier detection method. This metric captured periods of extremely high activation in traders.

Activation Length We analyzed the length of periods of continuous activation, which gave us information about the recovery time from periods of high activation, which in turn gave us some idea about the well being of the trader. To calculate the activation length, we followed the following procedure: after determining the periods of high activation, we calculated the length of each continuous period. If the periods of high activation were 5 minutes apart or more, they were considered different activation periods. For each trader on a given day, we calculated the median of the length of different activation periods to calculate the activation length.

Average Activation We calculate the mean value of activation over the day for a trader to obtain the average activation. The traders having higher activation proportion or length will have high average activation. Hence average activation captures the information from both the metrics activation proportion and length.

G. Traders Feedback Session

Initially we invited all 55 traders who participated in our study to the individual feedback sessions. Due to their busy schedule and limited availability, 14 of the 55 traders signed up for the feedback sessions. Each feedback session is conducted in a standardized format. For each trader, we created a report which summarized our PP activation analysis for this trader and shared the report with each trader the day before the meeting. The report positioned the PP activation level of the trader relative to the cohort of 55 traders in our study. It also included the market indices which have Granger-causal relation to the trader's activation levels. During the feedback session, we summarized the results and asked them the following questions:

- What causes your extreme activation levels during certain trading days (if there were any)?
- What market events and indices do you monitor on a regular basis?
- Are you interested in a follow-up study to analyze the relationship between your PP activation and trading performance (measured by PnL)?

H. Trader's Activation and Financial Product Type

The traders who participated in our study worked in different business divisions and traded a variety of financial products, including securitized markets (Securitized), credit, municipals (Muni), commodities, equities, foreign exchange and local markets (FXLM), and G10 rates. We investigate the relationship between financial products and trader activation measured by the regression coefficients.

We find that traders working in different business divisions had different average activation levels in the decreasing order: Commodities, FXLM, G10 Rates, Equities, Municipals, Credit and Securitized Markets. In table H, we present the regression coefficient of column business division compared to row business division along with the p-value and find the pairs of business divisions with statistically significant difference in their activation levels. For example, value $0.26_{0.04}$ in row *Securitized* and column *G10 R.* means that the difference between regression coefficients of G10 Rates product type and Securitized product type is 0.26 with p-value 0.04, which implies that G10 Rates traders had statistically significant higher activation than Securitized traders. The higher activation of FXLM and Equity traders can be attributed to high volatility in the markets that affects the PnL in real time while the G10 rate transactions were of higher amount (Table H) that can potentially lead to higher trader activation. We conclude that traders working in different business divisions have different profiles of PP activation.

Table A. Physiological waveforms measured by the Empatica E4 wristband for each trader. The IBI measures the length of the time interval between two successive heartbeats, and does not have a regular frequency.

**Physiological Waveform**	**Frequency**	**Unit**
Heart Rate (HR)	1	Number of beats
Blood Volume Pressure (BVP)	64	Millimeters of Mercury (mmHg)
Electrodermal Activity (EDA)	4	Microsiemens (μS)
Skin Temperature (TEMP)	4	Celsius (°C)
Wrist Accelerations (ACC)	32	$\frac{1}{32}g$
Inter-Beat Interval (ACC)	Irregular	Second(s)

Table B. Market time series data acquired from different data sources.

**Data Source**	**Market Data**
Chicago Mercantile Exchange	US Treasury Futures (2Y/5Y/10Y)
	E-mini S&P 500 Futures
	Crude Oil Futures
Global Financial Institution	US Dollar Index
	Credit-Default Swap Indices (HY and IG)
Chicago Board Options Exchange	VIX Futures

Table C. Total number of transactions made by the traders in each week. There is significant variation in the number of transactions across different weeks. Maximum transactions are concentrated in week 5 due to the use of automatic trading algorithms by traders.

**Total**	**Week 1**	**Week 2**	**Week 3**	**Week 4**	**Week 5**	**Week 6**	**Week 7**
51,732	145	308	305	1,577	45,246	3,523	628

Table D. Number of transactions before and after aggregating the block transactions for few representative traders on different days. The number of block transactions (column 4) is much closer to the number of transactions self-reported by the traders (column 5) than the raw data (column 3).

Trader ID	Date	Number of Trade	Number of Block Trade	Trader's Survey Response
A01A22	April 8	180	52	20
	April 9	279	14	20
	April 12	305	13	20
A020A2	April 29	64	22	15-50
	April 30	80	41	15-50
	April 12	305	13	15-50
	May 1	43	15	15-50
	May 2	110	75	15-50
	May 3	69	24	15-50
A02187	April 29	778	235	200-500
	April 30	1157	330	200-500
	May 2	946	233	200-500

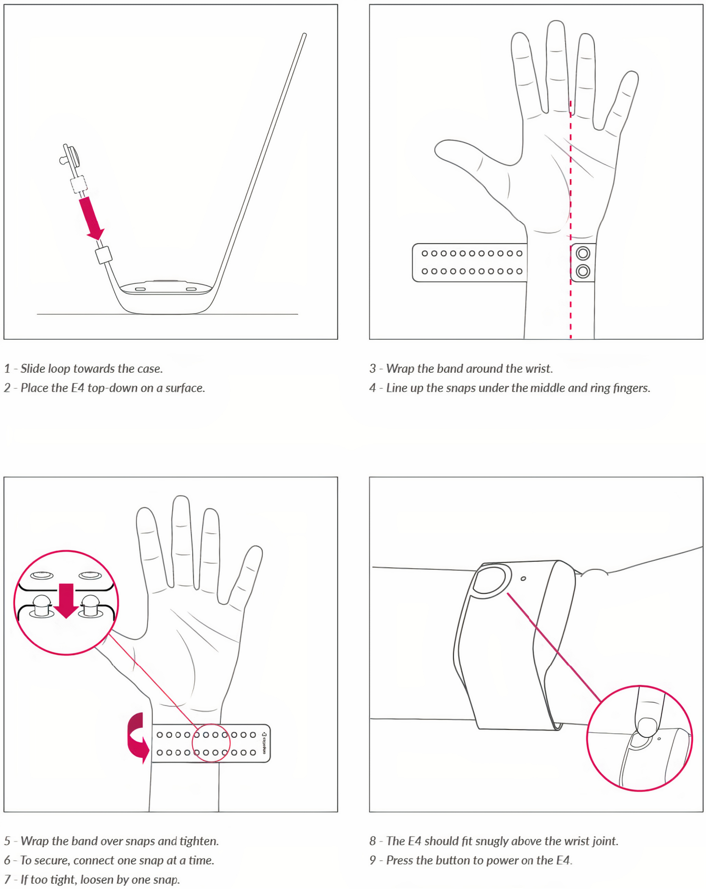

Fig A. Placement of the Empatica E4 wristband which measured traders' physiological waveforms (Table A). This figure was shown to the traders to illustrate how to wear the Empatica E4 wristband on their non-dominant-side wrist.

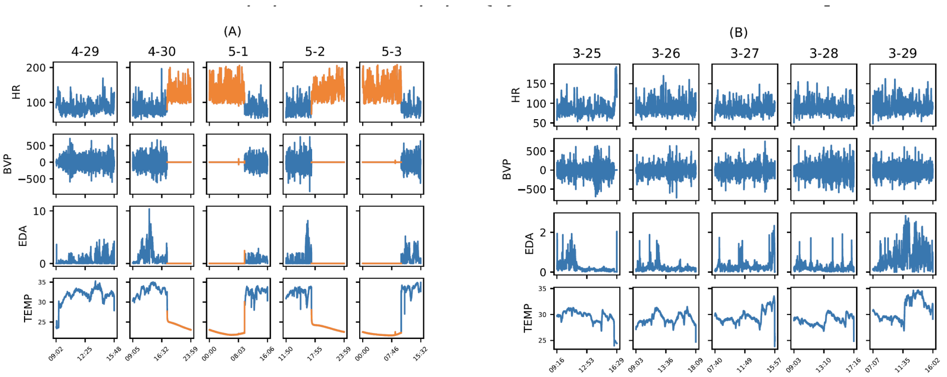

Fig B. Inactive periods in traders' physiological waveforms. (A) waveforms with inactive periods (plotted in orange). Based on the timestamps of the inactive periods, we suspect that this trader took off the wristband after work but did not terminate the measurements on 2019/4/30 and 2019/5/2. (B) waveforms with no inactive periods.

Table E. Trader Activation Statistics.

Trader Number	Average Act. (Mean)	Average Act. (Max)	Average Act. (Min)	Mild Act. Proportion (Mean)	Mild Act. Proportion (Max)	Mild Act. Proportion (Min)	Extreme Act. Proportion (Mean)	Extreme Act. Proportion (Max)	Extreme Act. Proportion (Min)	Act. Length (Mean)	Act. Length (Max)	Act. Length (Min)
1	4.04	4.91	2.77	12.62	13.94	11.29	3.68	3.83	3.53	131	214	41
2	3.70	5.09	3.07	10.25	10.78	9.92	2.30	2.69	1.66	126	159	91
3	4.17	5.34	3.70	11.82	14.97	9.08	1.96	2.77	0.76	94	124	78
4	3.80	4.54	3.18	11.38	13.87	8.95	2.91	4.11	1.92	161	182	126
5	3.78	4.79	3.45	11.00	13.59	8.48	2.50	3.82	1.15	125	232	62
6	3.75	4.54	3.21	11.91	14.00	10.17	2.37	3.13	1.33	126	228	76
7	3.79	4.31	3.13	10.33	11.61	9.83	2.86	5.15	0.62	158	281	70
8	3.97	4.54	3.41	13.62	13.62	13.62	3.37	3.37	3.37	134	218	48
9	3.96	4.32	3.32	11.51	13.34	9.45	3.65	4.93	2.62	142	222	38
10	4.03	4.45	3.48	12.32	14.66	11.42	3.62	4.43	1.93	266	338	110
11	3.59	4.00	3.07	11.75	14.71	9.58	2.68	4.52	1.40	182	269	106
12	3.79	4.11	3.19	11.74	16.04	9.66	2.94	4.25	2.01	197	346	112
13	3.78	4.18	3.35	11.24	16.58	8.75	2.85	3.44	1.96	168	293	98
14	3.77	4.17	3.34	12.47	15.08	9.66	2.14	3.83	0.51	195	278	86
15	3.79	4.31	3.48	11.30	13.39	10.50	3.15	6.22	0.80	308	1031	46
16	3.93	4.36	3.55	11.56	12.71	9.97	2.49	3.66	1.42	140	264	71
17	3.68	4.27	3.48	11.03	12.71	8.94	2.36	3.19	1.65	122	198	87
18	3.54	3.85	3.08	13.75	15.16	12.20	2.91	3.80	2.15	186	222	162
19	4.00	4.31	3.55	12.69	14.59	11.23	3.36	3.89	2.56	151	227	108
20	4.03	4.46	3.71	12.09	14.10	10.31	2.74	3.77	1.40	147	219	88
21	3.87	4.38	3.64	12.81	17.57	10.26	3.24	6.78	1.33	121	194	66
22	3.82	4.37	3.64	10.89	12.97	8.65	2.13	3.14	1.40	152	178	108
23	4.14	4.57	3.86	13.56	15.00	12.39	3.65	4.70	1.97	133	187	69
24	3.82	4.30	3.61	12.71	14.32	9.85	4.66	5.05	4.06	235	268	210
25	3.75	4.19	3.51	10.58	13.02	9.18	1.99	2.67	0.93	178	325	95
26	4.06	4.39	3.73	11.63	14.55	7.74	3.17	4.71	1.94	225	582	105
27	3.77	4.10	3.45	11.00	11.71	10.44	2.63	3.42	1.14	97	134	70
28	3.66	4.12	3.48	10.77	13.30	8.97	1.63	2.25	0.89	109	193	63
29	3.74	4.05	3.42	10.03	14.48	8.06	2.17	4.63	1.07	163	202	109
30	4.39	4.72	4.09	12.43	14.99	9.40	2.73	4.41	1.40	146	250	77
31	3.76	4.09	3.48	11.96	14.43	9.72	2.68	3.94	1.00	144	250	42
32	3.37	3.77	3.18	12.17	15.10	9.47	2.85	3.91	2.24	208	391	142
33	3.54	3.81	3.21	12.39	17.20	9.65	2.18	3.34	1.53	98	116	83
34	4.09	4.28	3.75	10.87	12.87	8.85	2.88	4.26	1.83	128	206	36
35	3.54	3.81	3.30	11.53	13.04	10.17	1.81	3.59	1.10	124	159	100
36	3.58	3.88	3.37	10.37	11.83	9.43	2.47	2.92	1.64	145	306	68
37	3.63	3.91	3.42	8.62	9.25	7.99	4.07	4.75	3.40	128	149	95
38	3.64	3.90	3.41	11.33	13.81	7.94	1.79	3.46	0.91	77	104	50
39	3.83	4.10	3.62	11.59	12.83	9.09	3.34	3.81	1.99	149	268	80
40	3.50	3.69	3.22	11.33	12.93	9.10	2.51	3.86	1.50	80	95	64
41	3.67	3.91	3.44	10.66	13.43	9.03	2.25	2.61	2.01	93	179	26
42	3.49	3.82	3.37	11.43	13.46	9.61	2.53	3.59	2.17	139	185	83
43	3.71	3.93	3.49	11.37	12.12	10.98	2.69	3.72	1.87	109	153	56
44	3.91	4.12	3.71	10.00	11.83	8.87	2.31	3.96	1.30	171	256	91
45	3.49	3.74	3.32	11.79	14.04	9.55	1.09	1.20	0.98	122	198	84
46	3.73	3.87	3.47	11.87	15.15	8.97	2.15	2.90	1.08	238	312	160
47	3.61	3.80	3.40	9.79	11.87	7.42	1.47	2.64	0.46	61	81	39
48	3.86	4.05	3.67	11.84	15.14	8.59	2.43	5.61	0.94	107	206	72
49	3.89	4.01	3.65	12.55	13.57	11.59	3.12	4.78	1.45	189	218	172
50	4.01	4.21	3.87	12.27	15.11	9.29	3.32	4.26	2.60	194	309	91
51	3.82	3.92	3.64	11.75	13.56	10.37	2.59	4.53	1.32	119	186	70
52	3.75	3.83	3.56	12.04	15.37	9.86	3.40	4.44	2.64	183	270	136
53	3.85	3.93	3.68	10.65	15.01	7.85	3.11	3.62	1.70	165	230	92
54	3.49	3.62	3.41	10.42	12.72	8.14	3.16	3.64	2.64	187	241	146
55	4.22	4.22	4.22	–	–	–	–	–	–	67	67	67

In the table above, we present the mean, maximum and minimum values calculated over different trading days for different activation metrics (average activation, mild activation proportion, extreme activation proportion, activation length). The values are sorted in descending order on the basis of difference between maximum and minimum average activation. The difference between minimum and maximum activation statistics signifies the varying activation for traders over days while difference between the rows signifies the varying activation across traders. Trader #55 had missing physiological data for multiple days potentially due to not wearing a wristband during trading hours. The units of mild and extreme activation proportion are percentages and the units of activation length are seconds.

Table F. In this table, we present the correlation values between different features that were used for average activation regression (refer heading *Activation Level Attribution Regression* under section *Materials and Methods* in main text). The higher values are shaded with red colour and lower values are shaded with blue. The number of transactions is positively correlated with experience implying experience traders places more number of transactions but the transactions were smaller in volume. While, use of automated trading algorithms led to higher number of transactions for equity traders. We also observed high correlation between G10 rate traders and dollar volume that can potentially explain the high activation in G10 rates traders. The activation level attribution regression was done for 194 different trader-days and similarly the correlation table was calculated using 194 different data points due to unavailability of trader's survey data for some traders. The p-values of the correlation are included in the brackets. The high positive correlation values are shaded in red and the high negative correlation values are shaded in green.

			Business									
		Experience	Commodities	Credit	Equities	FXLM	G10 Rates	Municipals	Securitized Markets	Female	Amount	NumTrade
	Experience	1.00(0.00)	0.24(0.00)	-0.27(0.00)	0.50(0.00)	-0.01(0.88)	-0.20(0.01)	-0.12(0.14)	-0.27(0.00)	-0.52(0.00)	-0.47(0.00)	0.62(0.00)
Business	Commodities	0.24(0.00)	1.00(0.00)	-0.15(0.08)	-0.21(0.01)	-0.15(0.07)	-0.19(0.02)	-0.11(0.20)	-0.15(0.07)	-0.25(0.00)	-0.36(0.00)	-0.17(0.04)
	Credit	-0.27(0.00)	-0.15(0.08)	1.00(0.00)	-0.20(0.01)	-0.14(0.08)	-0.18(0.03)	-0.10(0.23)	-0.14(0.08)	0.50(0.00)	0.07(0.41)	-0.17(0.04)
	Equities	0.50(0.00)	-0.21(0.01)	-0.20(0.01)	1.00(0.00)	-0.21(0.01)	-0.27(0.00)	-0.15(0.08)	-0.21(0.01)	-0.34(0.00)	-0.57(0.00)	0.72(0.00)
	FXLM	-0.01(0.88)	-0.15(0.07)	-0.14(0.08)	-0.21(0.01)	1.00(0.00)	-0.19(0.02)	-0.10(0.21)	-0.15(0.08)	0.21(0.01)	0.09(0.28)	-0.09(0.27)
	G10 Rates	-0.20(0.01)	-0.19(0.02)	-0.18(0.03)	-0.27(0.00)	-0.19(0.02)	1.00(0.00)	-0.13(0.11)	-0.19(0.02)	-0.01(0.94)	0.61(0.00)	-0.17(0.04)
	Municipals	-0.12(0.14)	-0.11(0.20)	-0.10(0.23)	-0.15(0.08)	-0.10(0.21)	-0.13(0.11)	1.00(0.00)	-0.10(0.21)	-0.05(0.55)	0.06(0.49)	-0.13(0.12)
	Securitized Markets	-0.27(0.00)	-0.15(0.07)	-0.14(0.08)	-0.21(0.01)	-0.15(0.08)	-0.19(0.02)	-0.10(0.21)	1.00(0.00)	0.03(0.73)	0.17(0.04)	-0.18(0.03)
	Female	-0.52(0.00)	-0.25(0.00)	0.50(0.00)	-0.34(0.00)	0.21(0.01)	-0.01(0.94)	-0.05(0.55)	0.03(0.73)	1.00(0.00)	0.19(0.02)	-0.26(0.00)
	Amount	-0.47(0.00)	-0.36(0.00)	0.07(0.41)	-0.57(0.00)	0.09(0.28)	0.61(0.00)	0.06(0.49)	0.17(0.04)	0.19(0.02)	1.00(0.00)	-0.44(0.00)
	NumTrade	0.62(0.00)	-0.17(0.04)	-0.17(0.04)	0.72(0.00)	-0.09(0.27)	-0.17(0.04)	-0.13(0.12)	-0.18(0.03)	-0.26(0.00)	-0.44(0.00)	1.00(0.00)

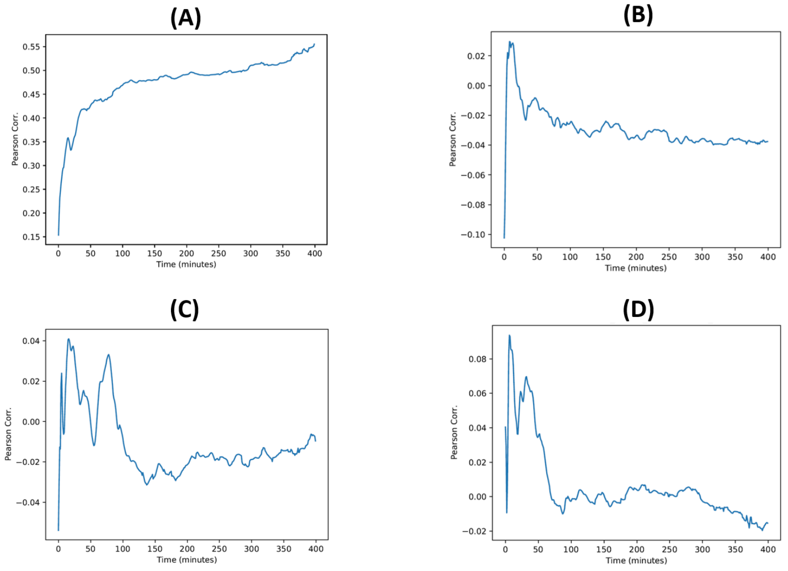

Fig C. Examples of rolling correlation between two physiology signals averaged over all traders. (A) EDA mean vs. EDA slope; (B) EDA mean vs.HR RMSSD; (C) EDA mean vs. HR mRRi; (D) EDA mean vs. BVP mean.

Table G. Correlation matrix between physiology signals averaged over all traders. The p-values (shown in parenthesis) are associated with the one sample t-test with the null hypothesis that the average correlation is zero for a pair of physiology signals. The statistically significant correlations (p-value < 0.05) are color coded.

	EDA mean	EDA slope	EDA events	BVP mean	BVP min	BVP max	HR mRRi	HR mHR	HR RMSSD	HR SDNN	TEMP slope
EDA mean	1.00 (0.00)	0.56 (0.00)	0.48 (0.00)	-0.01 (0.36)	0.02 (0.11)	-0.02 (0.16)	-0.02 (0.25)	0.01 (0.41)	-0.03 (0.03)	-0.01 (0.20)	0.01 (0.14)
EDA slope	0.56 (0.00)	1.00 (0.00)	0.87 (0.00)	0.15 (0.00)	-0.10 (0.00)	0.10 (0.00)	-0.10 (0.00)	0.09 (0.00)	-0.04 (0.00)	-0.01 (0.19)	-0.00 (0.99)
EDA events	0.48 (0.00)	0.87 (0.00)	1.00 (0.00)	0.19 (0.00)	-0.12 (0.00)	0.12 (0.00)	-0.12 (0.00)	0.10 (0.00)	-0.05 (0.00)	-0.01 (0.23)	0.00 (0.69)
BVP mean	-0.01 (0.36)	0.15 (0.00)	0.19 (0.00)	1.00 (0.00)	-0.52 (0.00)	0.53 (0.00)	-0.16 (0.00)	0.14 (0.00)	-0.01 (0.37)	0.05 (0.00)	-0.02 (0.05)
BVP min	0.02 (0.11)	-0.10 (0.00)	-0.12 (0.00)	-0.52 (0.00)	1.00 (0.00)	-0.75 (0.00)	0.07 (0.00)	-0.07 (0.00)	-0.04 (0.00)	-0.06 (0.00)	0.01 (0.07)
BVP max	-0.02 (0.16)	0.10 (0.00)	0.12 (0.00)	0.53 (0.00)	-0.75 (0.00)	1.00 (0.00)	-0.07 (0.00)	0.06 (0.00)	0.03 (0.01)	0.05 (0.00)	-0.02 (0.03)
HR mRRi	-0.02 (0.25)	-0.10 (0.00)	-0.12 (0.00)	-0.16 (0.00)	0.07 (0.00)	-0.07 (0.00)	1.00 (0.00)	-0.98 (0.00)	0.16 (0.00)	-0.11 (0.00)	0.05 (0.00)
HR mHR	0.01 (0.41)	0.09 (0.00)	0.10 (0.00)	0.14 (0.00)	-0.07 (0.00)	0.06 (0.00)	-0.98 (0.00)	1.00 (0.00)	-0.10 (0.00)	0.19 (0.00)	-0.05 (0.00)
HR RMSSD	-0.03 (0.03)	-0.04 (0.00)	-0.05 (0.00)	-0.01 (0.37)	-0.04 (0.00)	0.03 (0.01)	0.16 (0.00)	-0.10 (0.00)	1.00 (0.00)	0.68 (0.00)	-0.01 (0.39)
HR SDNN	-0.01 (0.20)	-0.01 (0.19)	-0.01 (0.23)	0.05 (0.00)	-0.06 (0.00)	0.05 (0.00)	-0.11 (0.00)	0.19 (0.00)	0.68 (0.00)	1.00 (0.00)	-0.03 (0.00)
TEMP slope	0.01 (0.14)	-0.00 (0.99)	0.00 (0.69)	-0.02 (0.05)	0.01 (0.07)	-0.02 (0.03)	0.05 (0.00)	-0.05 (0.00)	-0.01 (0.39)	-0.03 (0.00)	1.00 (0.00)

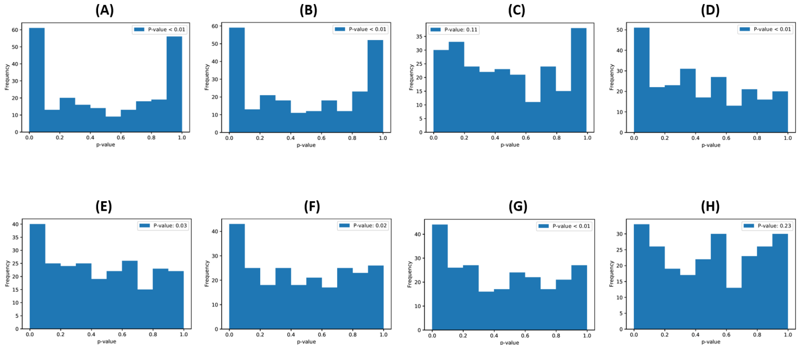

Fig D. Histograms of p-values of Granger causality tests between each market index and overall activation for all traders (using a lag time of 10 minutes). For each histogram, we perform a one sample KS test with the null hypothesis that the p-values are uniformly distributed between 0 and 1 (i.e., no statistically significant Granger causality relations). (A) Credit Default Swap Index IG; (B) Credit Default Swap Index HY; (C) USD Index; (D) S&P 500 E-mini Futures Price; (E) 10Y US Treasury Futures Price; (F) 5Y US Treasury Futures Price; (G) Crude Oil Futures Price; (H) VIX Futures Price

Table H. Regression coefficient of trader activation in the column business division with row business division as the baseline and the associated p-value.

**Business**	**Credit**	**Muni.**	**Equity**	**G10**	**FXLM**	**Commodity**
Securitized	$0.00_{,0.99}$	$0.07_{,0.70}$	$0.21_{,0.26}$	$0.26_{,0.04}$	$0.30_{,0.13}$	$0.30_{,0.07}$
Credit		$0.07_{,0.70}$	$0.21_{,0.28}$	$0.25_{,0.06}$	$0.29_{,0.11}$	$0.30_{,0.10}$
Muni.			$0.14_{,0.48}$	$0.18_{,0.30}$	$0.22_{,0.14}$	$0.23_{,0.24}$
Equity				$0.04_{,0.81}$	$0.08_{,0.66}$	$0.09_{,0.58}$
G10 Rates					$0.04_{0.82}$	$0.05_{,0.78}$
FXLM						$0.01_{,0.96}$

References

1. Sharpe WF. Efficient capital markets: a review of theory and empirical work: discussion. The Journal of Finance. 1970;25(2):418–420.

2. Lo AW. The Adaptive Markets Hypothesis. The Journal of Portfolio Management. 2004;30(5):15–29. doi:10.3905/jpm.2004.442611.
